# Pazopanib-induced enteritis in a patient with renal cell carcinoma

**DOI:** 10.1007/s12328-024-01919-w

**Published:** 2024-02-26

**Authors:** Misa Ariyoshi, Ryohei Hayashi, Takeshi Takasago, Ken Yamashita, Yuichi Hiyama, Ryo Yuge, Yuji Urabe, Yoshitaka Ueno, Fumio Shimamoto, Shiro Oka

**Affiliations:** 1 Department of Gastroenterology, Hiroshima University Hospital, 1-2-3, Kasumi, Minami-Ku, Hiroshima, 734-8551 Japan; 2 Department of Gastrointestinal Endoscopy and Medicine, Hiroshima University Hospital, 1-2-3, Kasumi, Minami-Ku, Hiroshima, 734-8551 Japan; 3 Faculty of Health Sciences, Hiroshima Cosmopolitan University, 5-13-18, Ujinanishi, Minami-ku, Hiroshima, 734-0014 Japan

**Keywords:** Pazopanib, Tyrosine-kinase inhibitor, Drug-induced enteritis, Dasatinib, Enteritis

## Abstract

A 69-year-old woman presented to our department with the chief complaint of diarrhea. She had undergone left nephrectomy for renal cancer 14 years earlier. Three years earlier, metastasis was detected in the left retroperitoneal cavity, and pazopanib administration was initiated. In the 29th month after the start of chemotherapy, the patient developed diarrhea, and on the 31st month, computed tomography showed thickening of the intestinal wall. Colonoscopy revealed white villi, intramucosal hemorrhage in the terminal ileum, and rough inflammatory mucosa with inflammatory polyps extending from the transverse to the sigmoid colon. Suspecting pazopanib-induced enteritis, we discontinued the medication, and the diarrhea resolved within 3 days. On the 21st day after discontinuation, colonoscopy revealed that the inflammatory polyps had shrunk, and the inflammatory findings had improved. Biopsy of the white villi of the ileum revealed histiocytes. The patient resumed treatment with pazopanib at 400 mg/day and developed soft stool on the 7th day after resumption. Compared with other tyrosine-kinase inhibitor-induced enteritis cases, this case showed less bleeding and more extensive inflammatory findings. There are similarities as well as differences from cases of previously reported pazopanib-induced enteritis. The mechanisms and characteristics of this disease require further investigation.

## Introduction

Pazopanib is a tyrosine-kinase inhibitor (TKI) of vascular endothelial growth factor receptor (VEGFR), platelet-derived growth factor receptor (PDGFR), and stem cell-derived growth factor (c-kit). Pazopanib is indicated for the treatment of renal cell carcinoma and malignant soft tissue tumors and was approved in the USA in 2009, ahead of the rest of the world, and in Japan in 2012. Diarrhea is a common adverse event caused by TKIs; for example, dasatinib causes diarrhea in 24.2% and sunitinib in 55% of cases. Pazopanib has been reported to cause diarrhea in more than half of the patients, 58% in a global Phase III trial for malignant soft-tissue tumors [1], and 63% in a global Phase III trial for unresectable or metastatic renal cell carcinoma [2]. However, there are only a few reports on its endoscopic findings. Immune checkpoint inhibitors, which have attracted attention recently, have been reported to cause diarrhea in 11–19% and 23–33% of cases during PD-1/PD-L1 inhibitor and CTLA-4 inhibitor therapy, respectively, and TKIs induce diarrhea at the same or higher frequency compared to these drugs.

Herein, we report a case of pazopanib-induced enteritis with endoscopic and pathological findings that changed over time. We compared this case with previous reports of enteritis induced by pazopanib or other TKIs and discussed the characteristics and pathogenesis of the disease.

## Case presentation

A 69-year-old woman presented to our department with the chief complaint of diarrhea. She had undergone left nephrectomy for left renal cancer 14 years prior. Six years prior, metastasis was detected inside the left abdominal wall, and the patient underwent tumor resection. Thereafter, the tumors recurred in the pelvis, retroperitoneum, and subcutaneous tissues; each time, the patient underwent surgery. Three years prior, the tumor had recurred in the left retroperitoneal space, and pazopanib was administered as systemic chemotherapy. The initial dose was 400 mg/day. The therapeutic effects were followed by a partial response to the progressive disease. The dose was increased to 600 mg/day in the 6th month after the start of chemotherapy and to 800 mg/day in the 12th month when the tumor was found to be enlarged. She had diarrhea since the 29th month, and abdominal contrast-enhanced computed tomography (CT) in the 31st month showed thickening of the intestinal wall; therefore, she was referred to our department.

**Fig. 1:**
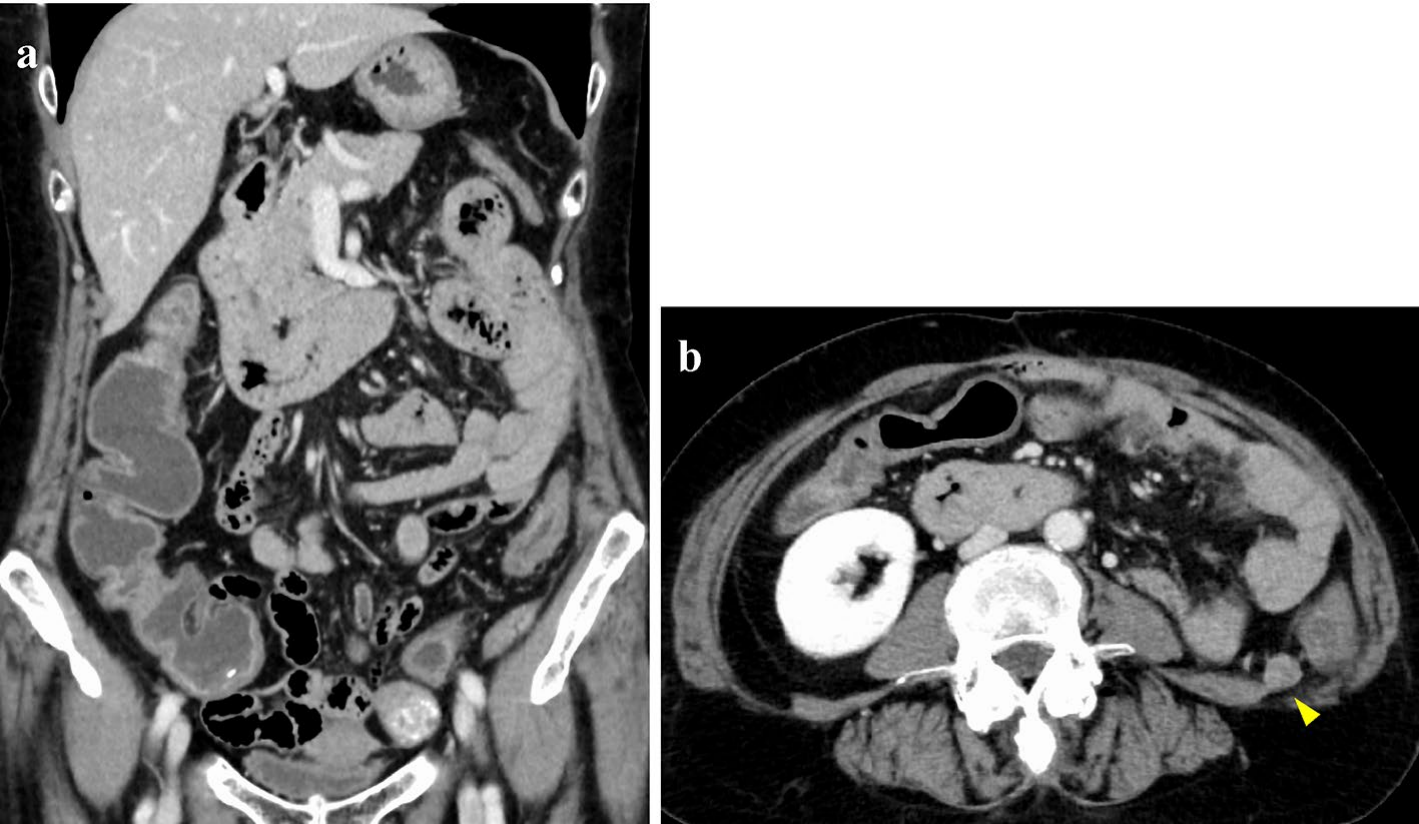
Contrast-enhanced abdominal computed tomography (CT) when diagnosed with pazopanib-induced enteritis. (**a**) Coronal section showing diffuse wall thickening of the colon. (**b**) Horizontal section showing metastases of the renal cancer in the left retroperitoneal cavity (arrowhead)

**Fig. 2:**
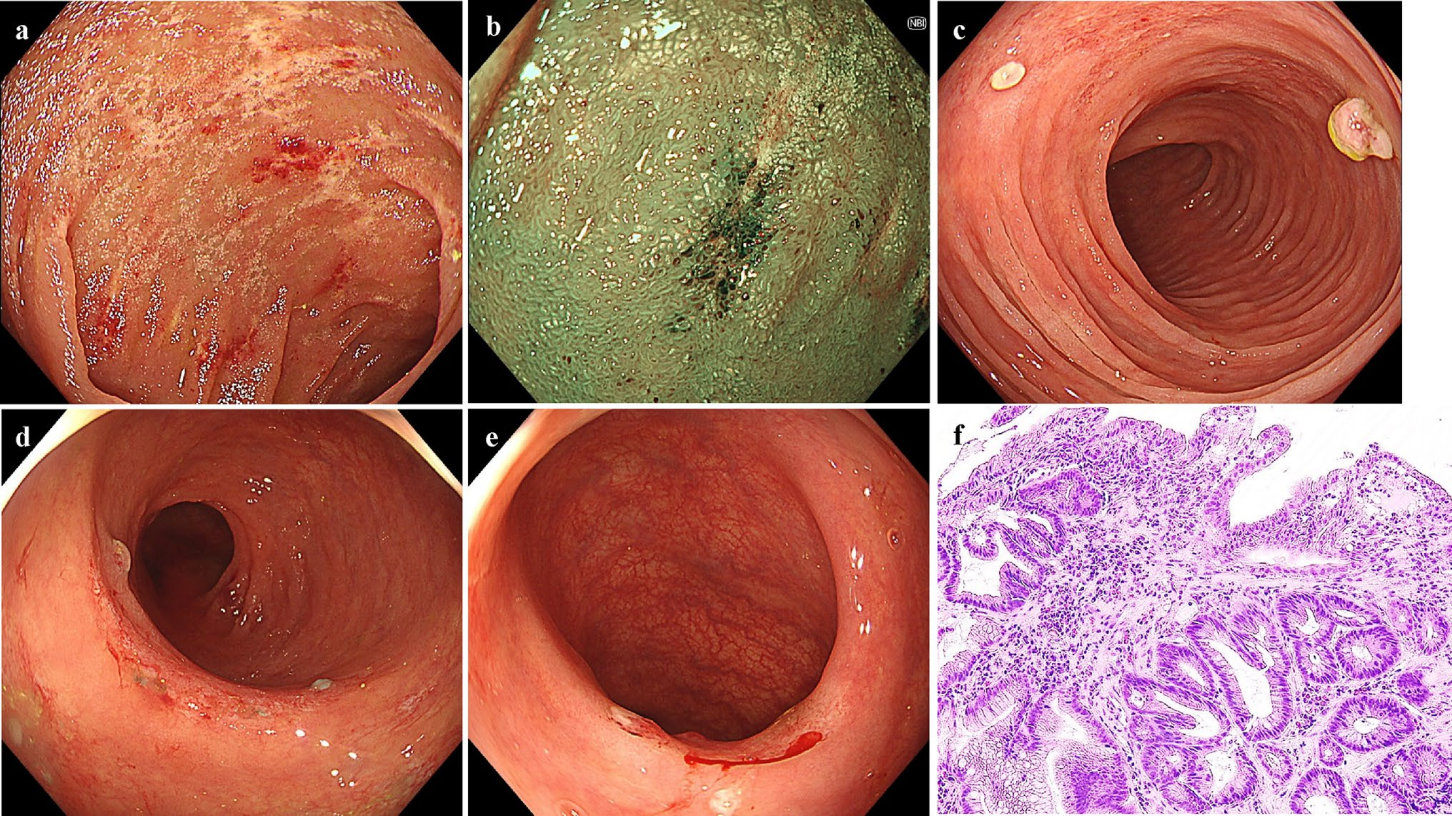
Colonoscopic and histopathological findings before drug discontinuation (**a**–**e**). Colonoscopic findings of the patient with pazopanib-induced enteritis. White villi, intramucosal hemorrhage, and erosion are observed in the terminal ileum (**a**). These are more prominent when viewed using narrow-band imaging (NBI) (**b**). Rough inflammatory mucosa with inflammatory polyps is observed in the transverse colon (**c**), descending colon (**d**), and sigmoid colon (**e**). (**f**) Histopathological findings of the transverse colon biopsies Inflammatory cell infiltration into the mucosal lamina propria, irregular branching of the crypts, atrophies, and hyperplastic changes are observed

**Fig. 3:**
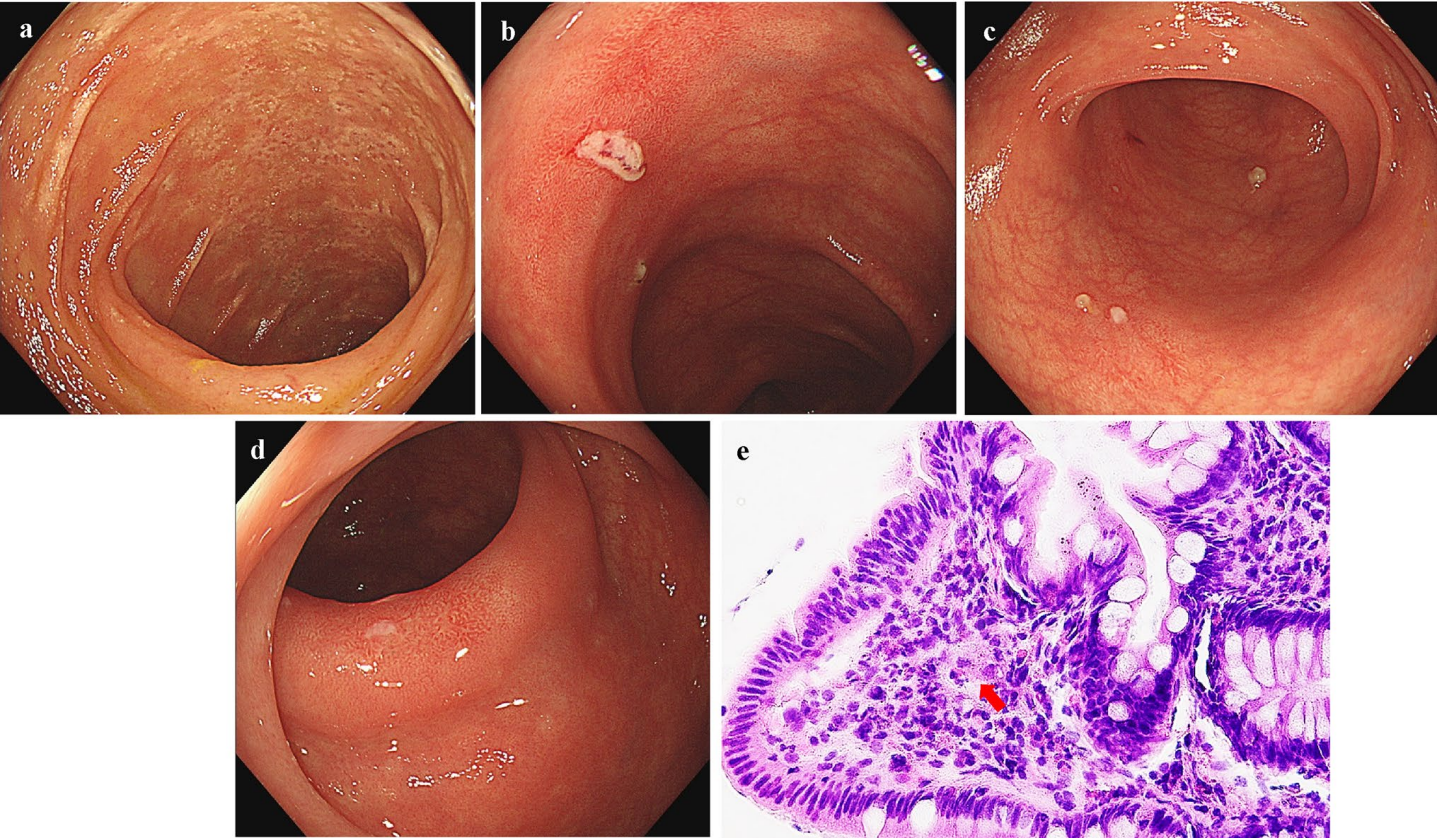
Colonoscopic and histopathological findings after drug discontinuation (**a**–**d**). Colonoscopy on day 21 after drug discontinuation. White villi persist, but the intramucosal hemorrhage and erosion disappear in the terminal ileum (**a**). Inflammatory findings improved, and inflammatory polyps shrunk in the transverse colon (**b**), descending colon (**c**), and sigmoid colon (**d**). (**e**) Histopathological findings of a biopsy specimen of the white villi of the terminal ileum Histiocytes with a crystalline material in the cytoplasm are shown (arrow)

**Fig. 4:**
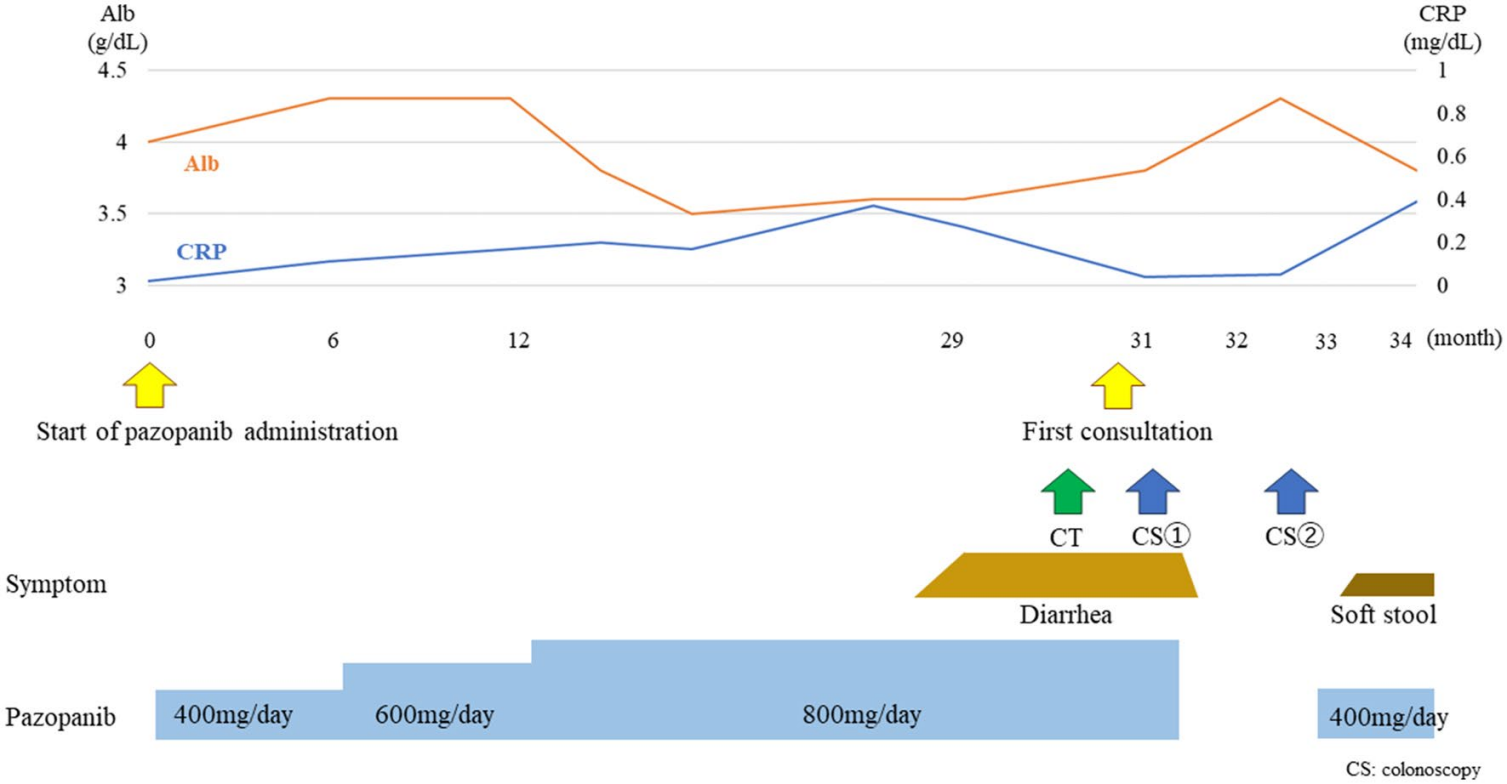
The progress chart after the start of pazopanib administration

She had no history of alcohol consumption or smoking. Comorbidities included hypertension and hyperuricemia. She had a medical history of cerebral infarction and adhesive intestinal obstruction. There was no history of allergic disease. She was on regular treatment with febuxostat, valsartan, amlodipine, aspirin, and furosemide. At the initial visit, her vital signs were stable, and she had no abdominal pain. She had watery diarrhea, and the defecation frequency was approximately three times a day. Blood tests showed no evidence of inflammation, and the serum albumin level was mildly low at 3.8 g/dL, with no other abnormal findings. Stool cultures revealed no pathogenic bacteria. Abdominal contrast-enhanced CT showed metastasis of the renal cancer in the left retroperitoneal cavity and extensive wall thickening of the colon (Fig. 1). Colonoscopy revealed white villi, intramucosal hemorrhage, erosions in the terminal ileum, and coarse inflammatory mucosa with inflammatory polyps extending from the transverse to the sigmoid colon (Fig. 2a–e). Histopathological biopsies showed inflammatory cell infiltration in the mucosal lamina propria, hyperplastic changes in the glandular ducts, irregular alignment, and atrophy from the ascending to the sigmoid colon (Fig. 2f). Based on the clinical course and various test results, pazopanib-induced enteritis was suspected, and the medication was discontinued. Diarrhea resolved within 3 days. On the 21st day after treatment discontinuation, blood tests showed that the serum albumin level had recovered to 4.3 g/dL and was within the normal range. Colonoscopy showed that the white villi in the terminal ileum persisted, although they were slightly less evident than before discontinuing the drug. However, the intramucosal hemorrhage and erosions disappeared. In the colon, inflammatory findings improved, and the inflammatory polyps had shrunk in size (Fig. 3a–d). Biopsy of the white villi of the terminal ileum revealed histiocytes with crystalline material in the cytoplasm (Fig. 3e). Endoscopic findings after treatment discontinuation showed an improvement in inflammatory findings, and a diagnosis of pazopanib-induced enteritis was made. The patient resumed treatment at 400 mg/day and began to have soft stools on the 7th day after resumption of treatment. Blood tests showed that the serum albumin level had decreased again to 3.8 g/dL. Because the symptoms were tolerable without the use of antidiarrheal agents, we decided to prioritize the treatment of the primary disease and continued the administration of pazopanib 400 mg/day. Figure 4 shows the progress chart after the start of pazopanib therapy.

## Discussion

Although diarrhea is a frequent adverse event associated with TKIs in general, there are only a few case reports that include endoscopic findings. Case reports of TKI-induced enteritis with endoscopic and histopathological findings are presented in Table 1 [3–10]. In most cases, endoscopic findings were nonspecific, such as erosions, erythema, and edema, and histologic findings were not characteristic. Some reports have suggested that inflammatory polyps are characteristic findings of TKI-induced enteritis (Table 1, case 4) [6], and in the present case, inflammatory polyps were also observed, which shrank after the drug was discontinued.

**Table 1:** Case reports including endoscopic images and histopathological findings of TKI-induced enteritis

Case	Year	References	TKI	Age (years)	Sex	Symptom	Period of medication to diagnosis	Location of inflammation	Endoscopic images	Histopathological findings
1	2013	Chisti [3]	Dasatinib	47	F	Bloody diarrhea	9 M	T/C–R	Congestive mottled erythema and microgranular mucosa	Lymphocyte infiltration to the lamina propria Lymphoid aggregates in the epithelium
2	2016	Miyazawa [4]	Dasatinib	74	M	Bloody stool	2 M	S/C–R	Multiple aphtha-like erosions with light yellowish exudate adherence and bleeding	S/C: inflammatory cell infiltration to the lamina propria Cryptitis Fibrin and necrotic material with neutrophil infiltration on the surface
3	2016	Yim [5]	Dasatinib	54	M	None (Anemia)	1 M	Whole colon	Multiple shallow ulcers with exudate and redness	Lymphocyte infiltration to the lamina propria The structure of the crypts was preserved
4	2018	Kobayashi [6]	Dasatinib	51	M	None (FOB)	2Y2M	T/C–R	Reddish multiple polyps (more than 100) with erosions and lichen white at the apex	Polyps: hyperplastic glands with cystic dilatation and smooth muscle hyperplasia
5	2018	Perdigoto [7]	Dasatinib	54	M	Bloody diarrhea	2Y6M	Whole colon	Loss of the normal submucosal vascular pattern, edema, erythematous, papular lesions associated with erosions, small ulcers, and exudation	Mild irregular and branched crypts, diffuse lamina propria hemorrhage, infiltration of eosinophils and neutrophils, and eosinophilic crypt abscesses
6	2021	Oshima [8]	Dasatinib	56	F	None (FOB)	1Y	T/C–D/C	Multiple erosions with yellow exudate and congested erythematous lesions	Lymphocyte infiltration to the lamina propria Cryptitis
7	2022	Wang [9]	Osimertinib	59	F	Abdominal pain Bloody stool	8 M	Cecum–S/C	Diffuse edema, erythema and bleeding ulcers	Inflammatory cell infiltration and ischemic changes in the lamina propria ulcers
8	2023	Wu [10]	Pazopanib	73	F	Diarrhea Weight loss	3Y	Jejunum	CS: terminal ileum and colon were normal DBE: diffusely swollen white villi in the jejunum	Jejunum: mild lymphoplasmacytosis No acute inflammation
9		The present case	Pazopanib	69	F	Diarrhea	2Y7M	Ileum A/C–S/C	Ileum: white villi and intramucosal hemorrhage T/C–S/C: rough inflammatory mucosa with inflammatory polyps	Ileum: histiocyte with crystalline material in cytoplasm A/C–S/C: inflammatory cell infiltration into the lamina propria, irregular branching, atrophy, and hyperplastic changes of the crypts

*A/C* ascending colon, *T/C* transverse colon, *D/C* descending colon, *S/C* sigmoid colon, *R* rectum, *FOB* fecal occult blood, *CS* colonoscopy, *DBE* double balloon enteroscopy, *M* month, *Y* year, *F* female, *M* male, *TKI* tyrosine-kinase inhibitor

The majority of cases were due to dasatinib, and only one case of pazopanib-induced enteritis (Table 1, case 8) [10] is reported that included an endoscopic finding. The common features of the present case and the reported case were white villi in the small intestine and plasma cells on histopathological examination. However, the reported case presented with protein-losing enteropathy, the inflammation was limited to the upper jejunum, and the endoscopic findings of the white villi were different from those in the present case, suggesting lymphangiectasia in the reported case. Therefore, further case accumulation is required to determine white villi as a specific finding in pazopanib-induced enteritis. Endoscopy was not performed in another reported case of pazopanib-induced enteritis [11]. A 46-year-old woman was treated with pazopanib for hemangiosarcoma. She experienced vomiting and abdominal pain for several days after the pazopanib dose was increased from 400 to 600 mg/day. CT showed wall thickening throughout the small and large intestines, which was diagnosed as pazopanib-induced enteritis.

The differential diagnoses in this case include concomitant medications and intestinal tuberculosis. However, concomitant medications were not considered to be related to the present enteritis. Although sprue-like intestinal disease can be caused by the concomitant drug valsartan, diarrhea is mild for sprue-like intestinal disease, and the endoscopic findings are atypical, with no noticeable small intestinal villous atrophy within the observation range. In addition, the fact that the patient's symptoms immediately resolved after pazopanib was discontinued and resurfaced after pazopanib was reintroduced highly suggests pazopanib-induced enteritis. No concomitant medications seemed to have potentiated the effect of pazopanib in this patient.

Tests to exclude tuberculosis were not performed in this case. Inflammatory polyps of intestinal tuberculosis are characterized by a cluster of polyps with surrounding erythematous erosions. Although the inflammatory polyps in the present case are atypical, the presence of erosions with a ring-shaped tendency suggests that intestinal tuberculosis was a relevant differential, and interferon-gamma-releasing assay, mucosal culture, etc. should have been considered.

In the present case, the diarrhea improved 3 days after pazopanib was discontinued; therefore, pazopanib-induced enteritis was considered more likely. Among the cases presented in Table 1, cases no. 2 and 7 showed improvement in symptoms 1 day and 5 days after discontinuation, respectively.

In other TKI-induced enteritis cases, bleeding symptoms such as bloody stool and fecal occult blood were the main symptoms, and the extent of inflammation was mostly in the distal colon from the transverse colon to the rectum. In contrast, in the present case, diarrhea was the main symptom, and there were extensive inflammatory findings from the terminal ileum to the sigmoid colon. All three cases of pazopanib-induced enteritis, including the present case, showed inflammatory findings in the small intestine. However, the localization of inflammation and imaging findings differed in each case. When pazopanib-induced enteritis is suspected, endoscopy for the small intestine (capsule endoscopy, balloon-assisted endoscopy) may be useful.

There are only a few reports of endoscopic images despite the high incidence of diarrhea with pazopanib, which may be because diarrhea as an adverse event occurs too frequently and rarely causes bleeding. Therefore, endoscopy is not performed, and symptomatic treatment is used in many cases. In reported cases of drug-induced enteritis caused by other TKIs, endoscopy was performed because of bloody stools or occult fecal blood. Therefore, there are many case reports of dasatinib-induced enteritis, which tends to cause bleeding among TKIs.

There have also been many cases of TKI-induced diarrhea with no abnormal endoscopic findings. Ueda et al. performed sigmoidoscopy on ten patients who developed diarrhea during VEGFR-TKI (sunitinib and cediranib) administration and performed a biopsy of the sigmoid colon, comparing the endoscopic and histopathologic findings. Endoscopic findings in the sigmoid colon were normal in seven cases, impression from outside and ulcer (metastasis of renal cell carcinoma on biopsy) in one case, telangiectasia in one case, and inflammatory findings (swollen mucosa) in only one case. There was only one case of endoscopically swollen mucosa showing mild inflammatory findings (limited abnormalities in architecture, focal bleeding, and edema). The other patients showed normal findings [12].

Although the mechanism of diarrhea after TKI administration is unclear, it is thought to be a combination of various factors, such as increased intestinal motility and dysregulation of the intestinal immune system [13]. It is also possible that the inhibitory effect on angiogenesis via PDGFR and VEGFR inhibition triggers inflammation due to ischemia of the colonic mucosa [14]. However, there were no findings of ischemic colitis in this case or previous reports of pazopanib-induced enteritis.

One of the factors that may contribute to dasatinib-induced enteritis and lower gastrointestinal bleeding is that the drug is excreted in the feces, and the lower gastrointestinal tract is exposed to the drug [15, 16]; pazopanib is similarly a drug that is mainly excreted in the feces. In a phase I overseas study (VEG10004), pazopanib was excreted in the stool at approximately 82.2% and in the urine at 2.6% by 168 h post-dosing, with an average of 67% of the dose excreted in the stool in the unchanged form. In contrast, 19% of dasatinib was excreted in the stool in an unchanged form, indicating different pharmacokinetics.

In the present case, white villi were observed in the ileum, and histiocytes were phagocytosed of crystalline material on biopsy, in addition to scattered intramucosal hemorrhages and erosions before drug discontinuation. After discontinuation of the medication, the intramucosal hemorrhage and erosions disappeared, but white villi remained. The white villi in the present case resembled the endoscopic findings of lanthanum deposition. Moreover, it has been reported that biopsies from white villi in lanthanum deposition also show histiocytes that have phagocytosed acidophilic substances and that white villi remain even after discontinuation of medication [17, 18]. In addition, intramucosal hemorrhage, erosion, and inflammatory cell infiltration were observed upon histopathological examination of the biopsy specimens. Including the fact that the drug was excreted in the stool, it is suggested that the gastrointestinal tract might have been exposed to the drug, directly causing intestinal inflammation. However, this finding was observed only in the present case, and further case studies are needed to clarify the characteristics and pathogenesis of pazopanib-induced enteritis.

In conclusion, there have been only a few case reports of pazopanib-induced enteritis, and we were able to confirm the changes in symptoms and endoscopic findings over time in the present study. The mechanism of pathogenesis is suggested to be direct inflammation of the gastrointestinal tract caused by drug exposure; however, the mechanism is not clear, and further case accumulation is desirable.
